# Neuropsychological Development and New Criteria for Extrauterine Growth Restriction in Very Low-Birth-Weight Children

**DOI:** 10.3390/children8110955

**Published:** 2021-10-23

**Authors:** Leticia Alcántara, Cristina Fernández-Baizán, Lara González-García, Enrique García-López, Clara González-López, Jorge Luis Arias, Marta Méndez, Gonzalo Solís Sánchez

**Affiliations:** 1 Hospital Universitario Central de Asturias, Universidad de Oviedo, 33003 Oviedo, Spain; Leticia.alcantara@sespa.es (L.A.); laraglezg@gmail.com (L.G.-G.); garcial.enrique@gmail.com (E.G.-L.); claragonlo93@gmail.com (C.G.-L.); 2 Faculty of Psychology, Universidad de Oviedo, 33003 Oviedo, Spain; fernandezbcristina@uniovi.es (C.F.-B.); jarias@uniovi.es (J.L.A.); mendezlmarta@uniovi.es (M.M.)

**Keywords:** newborn, prematurity, growth, neurodevelopment, postnatal growth

## Abstract

Background: Controversy between short-term neonatal growth of very low birth-weight preterm (VLBW) and neurodevelopment may be affected by criteria changes of extrauterine growth restriction (EUGR). Objective: to determine if new EUGR criteria imply modifications in the relationship between old criteria and results of neuropsychological tests in preterm children. Patients and methods: 87 VLBW at 5–7 years of age were studied. Neuropsychological assessment included RIST test (Reynolds Intellectual Sctreening Test) and NEPSY-II (NE neuro, PSY psycolgy assessment) tests. The relationships between these tests and the different growth parameters were analyzed. Results: RIST index was correlated with z-score Fenton’s weight (*p* = 0.004) and length (*p* = 0.003) and with z-score IGW-21’s (INTERGRWTH-21 Project) weight (*p* = 0.004) and length (*p* = 0.003) at neonatal discharge, but not with z-score difference between birth and neonatal discharge in weight, length, and HC for both. We did not find a statistically significant correlation between Fenton or IGW-21 z-scores and scalar data of NEPSY-II subtasks. Conclusion: In our series, neonatal growth influence on neuropsychological tests at the beginning of primary school does not seem robust, except for RIST test. New EUGR criteria do not improve the predictive ability of the old ones.

## 1. Introduction

Brain development of newborns with very low birth weight (VLBW) depends largely on correct nutritional intake during the immediate postnatal period. Nutrition and, therefore, body and brain growth in the first weeks of life may be of great importance for the neuropsychological development of these children [1].

Postnatal growth rate of VLBW and its relationship with neuropsychological development has been studied by many authors with different conclusions [1,2,3,4,5,6,7,8,9]. Such result variabilities are given by the different growth parameters analysed, different neurodevelopmental tests used, and different ages studied. Indeed, neurodevelopment is influenced by many other factors, pre- and postnatal, organic (gestational age, Apgar score, newborn morbidity, brain injuries…) and social (early care, parent social level, education…) that can confuse or modify this relationship [10,11,12].

Nevertheless, several studies show that the neuropsychological development of preterm infants can affect functions, for instance, memory [13,14], visuospatial skills [15,16], or executive functions [17,18], although these have not been considered in previous literature with respect to postnatal growth.

When we analyse growth of premature infants in the first weeks of life, two important questions arise: what is the ideal growth of these infants and what measure is the most appropriate to verify it? [19,20,21].

Since 1977, the American Academy of Pediatrics recommended using foetal growth as an ideal postnatal growth in preterm infants [22]. For this reason, most hospitals have employed reference curves based on cross-sectional measurements of neonatal weight of neonates at birth with different gestational ages (GA). In the last 20 years, Olsen and Fenton’s charts [23,24,25], with their progressive modifications, have been basics in calculations and classifications of preterm infants, especially in high-income countries.

With these curves, neonates are classified in percentiles or z-scores, allowing to label neonates as small (IUGR), normal, or large for their GA, in weight, length, and cranial perimeter. Additionally, such curves are used to label children’s growth from birth, during their stay in neonatal units, until 28 days, 36 weeks of GA or discharge, in order to assess so-called extrauterine growth restriction (EUGR).

However, in recent years, several changes in these concepts have been postulated. It is advocated not to use curves created with data from cross-sections at birth, but rather ones that define how a healthy premature infant should grow, without prenatal or postnatal complications throughout their first months of life [21,26]. This reasoning has changed 1997 AAP’s (American Academy of Pediatrics) paradigm to a new approach based on standards such as those of INTERGROWTH-21st Project (IGW-21), which provides data of preterm infants’ growth from birth to week 64 of postmenstrual age [27,28]. Besides, these IGW-21 data couple with the WHO curves at this age (WHO Child Growth Standards [29]).

Articles have been published comparing both strategies, recalculating percentages of IUGR preterm infants (weight below the 10th percentile at birth) and static EUGR (weight below the 10th percentile at discharge, or at 36 weeks of corrected GA), differing according to graphs/standards used [30].

Another change that arises with this publication is whether we should call EUGR to all neonates who are below the 10th percentile at discharge or at 36 weeks of corrected GA, or only those who are born above this percentile (non-IUGR) and are below it at discharge, what some authors call true EUGR [31]. To end this discussion, other authors prefer to use the dynamic concept of loss of 1 or 2 standard deviation (SD) in the z-score from birth to discharge to speak of EUGR, what we could call dynamic EUGR.

In any case, debate remains open and discussion on the classification of premature children, with static or dynamic criteria, creates new fronts in the effect of growth on the neuropsychological development of these children [2].

For all the above, our aim was to analyse neuropsychological functions at 5–7 years, (beginning of primary education) in a cohort of children under 1500 g at birth, and to determine if new EUGR criteria imply modifications in the relationship between old criteria and results of neuropsychological tests in preterms. That is, if new criteria are more precise than the previous ones in labelling those cases with the lowest neuropsychological outcomes at 5–7 years of age.

## 2. Materials and Methods

An observational study was conducted, including 87 VLBW (less than 1500 g) who were born in the Neonatology Service of the Central University Hospital of Asturias (region of northern Spain, with one million inhabitants) between 1 January 2009 and 1 January 2012.

In this period, 181 initial cases were born in our hospital, of which 34 died before discharge and 147 survivors were invited to participate in this study, responding affirmatively 87 cases (51 males and 36 females). All of them were set a date for a clinical assessment a neuropsychological study in our hospital between February 2016 and 6 May 2017. Comparing the studied cases (87) and those that were not studied (60 lost cases), we did not find statistically significant differences neither in neonatal variables, nor in neurological evolution at medium-long term (Table 1).

In the 87 studied cases, the assessment protocol was completed with anthropometry at birth, anthropometry at discharge from neonatal admission, and anthropometry at 2 years of chronological age corrected for GA, all of them obtained from medical records. The birth and discharge data were analysed with Fenton and IGW-21 graphs and standards, while 2 years data was analysed with WHO graphs.

We have considered as IUGR those cases born below the 10th percentile for GA. We have defined static (or cross-sectional) EUGR as cases that were below the 10th percentile for GA at neonatal discharge. We have considered as true EUGR the cases that, have being born above the 10th percentile (not IUGR), were discharged below the 10th percentile for their GA. Finally, we have defined dynamic (or longitudinal) EUGR those cases that lost more than 1 or more than 2 SD from birth to discharge, regardless of their initial or final percentiles.

During the clinical assessment (study visit), a neuropsychological evaluation was performed by psychologists, including an estimation of IQ and an assessment of the inhibitory control capacity, the verbal and visuospatial memory and the visuospatial abilities, using the RIST test and four task from NEPSY-II battery.

The RIST (Reynolds Intellectual Screening Test) provides an IQ estimation score for children and adults between 3 and 94 years old by employing two tasks: “guess what” for the verbal score and “odd-item” for the non-verbal score. On “guess what” (verbal task), the examiner reads some definitions, and the child has to answer with the appropriate word. On “odd-item” (non-verbal task), the child is shown several pictures, and s/he has to point to the different or incongruent one. Its reliability coefficient varies between 0.89 and 0.91 depending on the age of child and the task [32].

The NEPSY II battery [11,33] allows us to obtain a neuropsychological profile in children from 3 to 16 years old. The following subtests were used in the present study: inhibition, memory for names, memory for designs, and route finding.

The “inhibition” subtest was used to measure inhibitory control. In this task, the child is shown several black and white geometric shapes (circle and square) or black and white arrows, and s/he has to say the correct (denomination) and opposite (inhibition) shape or direction of the arrow.

The “memory for designs” subtest was employed to assess short- and long-term visual and spatial memory. In the short-term assessment, the child is shown a grid containing from 4 to 10 meaningless picture cards per page. Then, the examiner removes the page, and the child must select the designs s/he has seen from a set of target cards and distractors and place them on a grid in the same location s/he was previously shown. This procedure is repeated 15–25 min later for the long-term evaluation.

The “memory for names” subtest was used for short- and long-term verbal memory evaluation. To assess short-term memory, the child is shown 6–8 cards containing pictures of children while the examiner reads the name of each child on the cards. Then, the cards are shown again, and the examinee is asked to remember the name of the child on each card. To assess long-term memory, the same test is applied after about 25–35 min.

The “route finding” subtest was employed for directionality, spatial relation, and map interpretation assessment. The child is shown a schematic map indicating how to reach a house. The child is then asked to find the house on a larger map with more roads and houses available. For the NEPSY-II battery, scalar scores from all these tests are reported.

Variables with normal distribution were compared with Student’s *t* test. We used non-parametric test for Birth Weight (Z Kolmogorov–Smirnov test 1.320, *p* = 0.06), and for neuropsychological test comparations, because these tests were value like scaled scores (Mann–Whitney U test). Comparisons of percentages were made with the Chi-square test (using Fisher’s exact test if expected were less than 5). To establish correlations between z-scores, Spearman Rho test was used. Finally, Kappa concordance test was used to compare Fenton and IWG-21 classifications. Statistical significance was maintained throughout the study for *p*-value < 0.05.

Parents were informed about the aims of the study and provided their written informed consent before the study began. The study was conducted in accordance with the Helsinki declaration for research in human subjects, and it was approved by the regional ethics committee (Research Ethics Committee of Principado de Asturias, Spain, study number 144/15).

## 3. Results

### 3.1. Description of the Growth of 87 Cases Studied

Anthropometric data at birth, at discharge from neonatal admission, and at 2 years of age are shown in Table 2, while Table 3 contains the classifications at birth, at neonatal discharge, and at 2 years according to Fenton, IGW-21, and WHO graphs and standards, with their Kappa concordance.

### 3.2. Description of Results of Neuropsychological Tests

**Figure 1:**
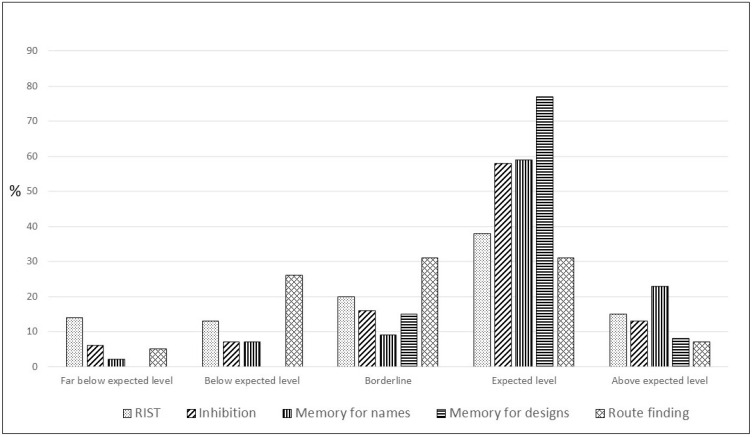
Results of neuropsychological assessment grouped into categories.

Median (interquartile range) IQ for RIST was 91 (78.7–101). Median scalar score (interquartile range) for Inhibition was 9 (7–11), for Memory for names was 10 (7–11), for Memory for designs was 10 (8–12), and for Route finding was 2 (1–4). In Figure 1, we can see the results of neuropsychological assessment grouped into categories.

Comparing scalar scores or IQ scores of different tests between cases with and without clinical neurological alterations diagnosed during development, we found statistically significant differences in Inhibition and in Route finding (Table 4).

### 3.3. Relationship between Growth and Neurological Clinical Development and Neuropsychological Tests

There were no significant associations between growth disturbances and to have a clinical neurological development disorder, except for the loss of 2 SD in length between birth and neonatal discharge for Fenton.

**Figure 2:**
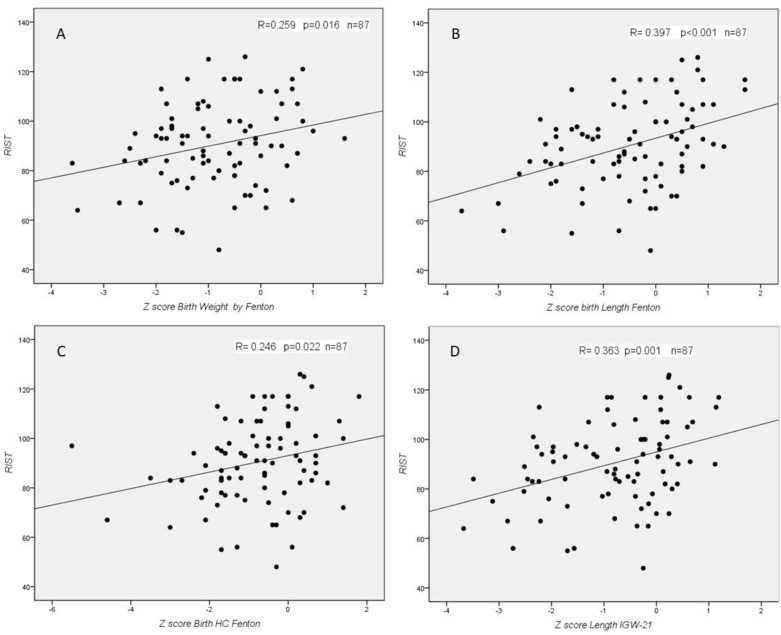
Correlations between RIST index and z-score for Fenton at birth (**A**: weight; **B**: length; **C**: and HC) and for IGW-21 at birth (**D**: length).

**Figure 3:**
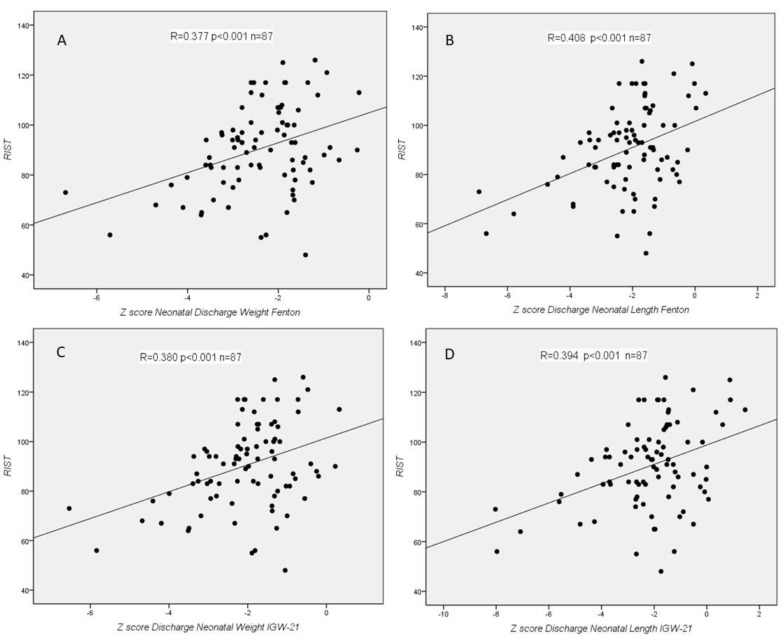
Correlations between RIST index and z-score for Fenton at discharge (**A**: weight, and **B**: length) and for IGW-21 at discharge (**C**: weight, and **D**: length).

We found statistically significant correlations between RIST index and z-score for Fenton at birth (weight, length, and HC), for IGW-21 at birth (length), for Fenton at discharge (weight and length), and for IGW-21 at discharge (weight and length) (Figure 2 and Figure 3). We did not find any statistically significant correlation between RIST and z-scores at 2 years, nor between RIST and z-score difference between birth and neonatal discharge. In the NEPSY-II test, we did not find any statistically significant correlation.

Comparing RIST index values between patients with or without IUGR or EUGR, we found some statistically significant differences (Table 5).

Finally, we found a statistically significant relative risk for having HC IUGR by Fenton and for obtaining RIST scores lower than 89 (RR 1.8 (CI 95% 1.2–2.9), and for having IUGR weight by Fenton or IUGR weight for IGW and to obtaining a percentile of 25 or lower in the Route finding subtask (RR 1.5 (CI 95% 1.1–2.0) and RR 1.5 (1.1–2.1), respectively).

## 4. Discussion

VLBW may be due to prematurity and/or IUGR of different etiologies. Later, growth of these neonates can be influenced by multiple genetic, epigenetic, neonatal (respiratory, cardiac, infectious, nutritional, etc.), and post-neonatal (nutrition, morbidity, culture, etc.) factors. On the other hand, the neurodevelopment of these children is also influenced by different factors, some of them coinciding with the abovementioned and some of them being related to other matters (parental intelligence, socio-family level, education, etc.). In published observational studies, it has been analysed whether growth is related to neurodevelopment in the short, medium and long term, although the conclusions are not uniform, and debate remains open [2,3,4,5,6,7,8,9].

Beyond major neurological alterations diagnosed clinically in the follow-up (motor, sensory, cognitive, behavioural…), VLBW may present neuropsychological alterations at the school age, only detectable by specific tests for this purpose, such as the RIST test and the NEPSY-II battery at 5–7 years. For this reason, our study aimed to know the relationship between the result of these tests and initial growth of VLBW, comparing different definitions of neonatal growth, without entering the influence of other neonatal factors that are already present in previous publications [11].

### 4.1. Growth

One of the main problems that arises when assessing the relationship between neonatal growth and subsequent neurodevelopment is which growth parameters and anthropometry to use. Classically, Fenton’s 10th percentile was cut-off at birth and neonatal discharge for classifying children as IUGR and EUGR, but this classification is changing. First, IGW-21 standards seem much more interesting as basic data, given the way they are obtained (longitudinal with healthy preterm infants), to classify children [27], although it is still not clear whether we should call all those under the 10th percentile at discharge or if we should only use this term for those who, born above the 10th percentile, go from discharge below it. Other authors go further, proposing that EUGR concept should be based on the reduction of standard deviations, rather than static percentiles, adding a dynamic and critical look to this discussion [27] This is how the concept of static (or transverse) EUGR and dynamic (or longitudinal) EUGR arose, the latter being the currently preferred [2,4,22,34,35].

Our study aimed to evaluate these aspects, analysing static anthropometry data (weight, length, and HC at birth and discharge), but also some dynamic ones (true EUGR and changes less or greater than −1 and −2 SD between birth and discharge), both for Fenton and IGW-21. Our results support that the percentage of VLBW classified as IUGR and EUGR varies greatly depending on the criteria used.

Thus, IGW-21 is slightly less selective at birth in IUGR classification (that is, IGW-21 labels more cases than Fenton as IUGR) but, at the same time, is stricter at discharge in the static EUGR classification for weight (IGW-21 labels less cases than Fenton as static EUGR). Thus, some true Fenton-tagged EUGRs are not correctly detected as such (30.4% in our series) by IGW-21, while Fenton detects all the IGW-21-tagged ones. However, this situation only happens with the weight measurement, but not with length nor with HC, which do not present a uniform trend, contrary to what appears in other series [36].

### 4.2. Neuropsychological Test

In our series, RIST scores, as a measurement of IQ, were correlated with z-score at birth (Fenton’s weight, length, and HC, and IGW-21’s length) and at neonatal discharge (Fenton and IGW-21 weight and length), but not with z-score differences between birth and neonatal discharge, nor with the z-score at 2 years. In addition, IUGRs in weight, length, and HC, and static EUGRs in length and HC show lower scores, statistically significant, compared to neonates who were not, with differences of 10–20 points between them (Table 5).

These results coincide partially with the previous literature, finding that some neonatal and early postnatal measures, which indicated a lower growth status, could be related with later and lower IQ achievements [2].

However, it is also worth mentioning some major methodological differences of our study with some previous publications, such as the use of different definitions of EUGR, with Fenton and IGW-21 graphs and standards. Therefore, looking at our results, we can say that the new approaches of growth classifications (the use of the IGW-21 standards or the new dynamic definitions) do not modify the findings of the classical criteria. IGW-21 standards do not improve the performance of Fenton graphs in terms of RIST prognosis, and the new EUGR concepts (dynamic or true) do not either.

Another issue to consider would be the different importance given to the IQ in these previous publications. While previous articles are focused on IQ assessment as the only measurement of cognitive development, our aim was to consider a wider neuropsychological profile, and due to that, the RIST test was included only as a screening measure of IQ. With these results, we must admit that IQ index is mathematically related with static somatometric values at birth and discharge, but not with dynamic ones. In any case, this relationship, as we discussed previously, can be influenced by many other prior or intermediate factors that surely play important roles.

In the NEPSY-II tests, we did not find a relationship with the growth parameters analysed. Perhaps this is because these subtasks are excessively specific, or because we have not gotten enough power with our sample size to find possible true relationships. It is striking, for example, that Memory for names and Memory for design tasks, related to verbal and visuospatial memory, respectively, did not present any type of relationship (neither correlation nor statistically significant differences) with any growth parameter. This may be since the memory scores reported in the present study are composite indexes of both short- and long-term memory, which are related but independent cognitive functions and their addition could decrease their ability to differentiate between growth statuses. Besides, it should be noticed that in Memory for designs, any of our participants scored below expected, and then, this can limit the discriminatory capacity of such a task. We also did not find correlations between scalar scores of other studied NEPSY-II subtasks (Inhibition and Route finding subtasks) and the z-scores of different measures. These findings could point to the possibility that slower growth does not affect specific neuropsychological functions, but rather a more global pattern of cognitive development.

Among the strengths of our study, we believe that we can point out that it is a prospective study, specifically designed to assess RIST and NEPSY-II in a cohort of children under 1500 g, in a university hospital. It is also a strength that the field study was carried out by only two psychologists and one paediatrician, with little or no variability in the assessment of the tests and the variables under study. Among the limitations, we can point out that the study was limited to the age of 5–7 years, an age in which these premature infants can still present significant intra-group variations in their brain maturation.

In the future, the advantages of using some graphs over others (IGW-21 vs. Fenton) and of some EUGR criteria over others (static, dynamic, or true) should be evaluated in further development studies, in order to determine which graph and which criteria are the best when it comes to predicting the neuropsychological future of these premature infants.

## 5. Conclusions

In summary, classification IUGR and EUGR (static, dynamic, and true) with Fenton and IGW-21 show very varied percentages according to the criteria used in the classification. Besides, growth influence on neuropsychological tests at 5–7 years of age does not seem robust, except for IQ measurement (RIST test), which is clearly correlated with some static measures, and which shows clinically important differences between some groups classified according to these measurements. However, the new criteria do not improve the predictive ability of the old ones, nor does IGW improve Fenton for this purpose.

Reviewing growth importance in the first weeks of life in later neurodevelopment is a topic of great interest to neonatologists, endocrinologists, neurologists, and psychologists. New IUGR and EUGR concepts should be evaluated in future studies in this field to know exactly its prognostic capacity in long-term neurodevelopment.

## Figures and Tables

**Table 1:** Comparison between studied cases and survivors not studied (lost) for the perinatal and neonatal variables.

	Studied Cases (*n* = 87)	Survivors Lost (*n* = 60)	Statistical Significance
Weight (g): median (IQR)	1220 (430)	1163 (413)	0.682 ^#^
Gestational age (weeks) mean (CI 95%)	29.6 (29.0–30.2)	29.8 (29.0–30.6)	0.774 ^&^
Sex: male/female (*n*)	51/36	31/29	0.404 ^@^
Multiple birth (*n*)	30	15	0.220 ^@^
Type of delivery: Vaginal/Caesarean (*n*)	27/60	23/37	0.359 ^@^
Apgar test (*n*)			
1 min: 0–3/4–6/>6	9/16/62	4/10/46	0.689 ^@^
5 min: 0–3/4–6/>6	1/5/81	2/0/58	0.115 ^@^
Neonatal resuscitation (*n*)			
Oxygen	74	49	0.585 ^@^
Ambu with mask	57	32	0.137 ^@^
CPAP nasal	26	18	0.737 ^@^
Intubation	35	18	0.204 ^@^
Heart massage and/or Drugs	7	2	0.188 ^@^
Respiratory support:			
Oxygen	78	51	0.397 ^@^
Non-invasive ventilation (IMVn)	46	33	0.799 ^@^
Conventional ventilation	47	37	0.357 ^@^
High frequency ventilation	2	2	0.705 ^@^
Diagnoses:			
Hyaline membrane disease	43	31	0.789 ^@^
Patent ductus arteriosus	21	16	0.728 ^@^
Necrotizing enterocolitis	4	1	0.335 ^@^
Early sepsis	1	2	0.357 ^@^
Transfusable anaemia	35	23	0.678 ^@^
Late sepsis	23	18	0.636 ^@^
Intracranial haemorrhage	21		
I or II	2	15	0.562 ^@^
III or IV		4	
Periventricular leukomalacia	9	6	0.774 ^@^
Retinopathy of prematurity			
I	13	8	0.985 ^@^
II or III	15	9	
Fundus not performed	4	3	
Neurological development:			
- Neurological disorders of any kind	19	14	0.831 ^@^
- Major neurological disorders	7	8	0.297 ^@^
- Congenital alterations not related	2	2	0.704 ^@^
- Cerebral palsy	5	5	0.540 ^@^
- Severe developmental disorders	3	2	0.969 ^@^
- Language disorders	12	4	0.172 ^@^
- Behavioural disorders and/or attention deficit hyperactivity disorder	3	2	0.969 ^@^

# = Mann–Whitney U-Test; & = T-Student; @ = Chi-square. Major neurological disorders: cerebral palsy or severe development disorder or blindness or deafness or epilepsy.

**Table 2:** Anthropometry at birth, neonatal discharge, and at 2 years of the 87 neonates followed.

	Weight (g)	Length (cm)	Head Circumference (cm)
Birth			
Mean (CI 95%)	1153 (1101–1204)	37.8 (37.1–38.5)	26.2 (25.7–26.7)
Median (IQR)	1220 (430)	38.0 (5)	27.0 (4)
Neonatal discharge			
Mean (CI 95%)	2325 (2277–2372)	45.4 (45.0–45.8)	33.2 (32.9–33.5)
Median (IQR)	2260 (225)	45.0 (2.5)	33.0 (1.5)
2 years			
Mean (CI 95%)	11403 (11030–11775)	86.0 (84.9–87.1)	48.4 (48.0–49.8)
Median (IQR)	11425 (2100)	86.0 (6.5)	48.5 (2.0)

**Table 3:** Classification at birth, neonatal discharge, and at 2 years, by Fenton, Intergrow-21, and WHO (*n*,%).

		Weight	Length	Head Circumference
Birth	IUGR			
	Fenton less than P10	33 (37.9%)	23 (26.4%)	29 (33.3%)
	IGW-21 less tan P10	34 (39.1%)	28 (32.2%)	34 (39.1%)
	Kappa concordance	0.879	0.807	0.826
Neonatal discharge	Static EUGR (cross-sectional)			
	Fenton less than P10	78 (89.7%)	68 (78.2%)	25 (28.7%)
	IGW-21 less than P10	66 (75.9%)	63 (72.4%)	28 (32.2%)
	Kappa concordance	0.532	0.785	0.81
	Fenton less than −2 SD	50 (57.5%)	41 (47.1%)	11 (12.6%)
	IGW-21 less than −2 SD	41 (47.1%)	42 (48.3%)	15 (17.2%)
	Kappa concordance	0.795	0.839	0.82
	True EUGR			
	For Fenton	46 (52.8%)	45 (51.7%)	9 (10.3%)
	For IGW-21	32 (36.8%)	38 (43.6%)	10 (11.5%)
	Kappa concordance	0.683	0.748	0.705
	Dynamic EUGR (logitudinal)			
	More than -1 SD Fenton	54 (62.1%)	56 (64.4%)	17 (19.5%)
	More than -1 SD IGW-21	41 (47.1%)	45 (48.3%)	17 (19.5%)
	Kappa concordance	0.705	0.605	0.781
	More than −2 SD Fenton	23 (26.4%)	26 (29.9%)	8 (9.2%)
	More than −2 SD IGW-21	19 (21.8%)	26 (29.9%)	8 (9.2%)
	Kappa concordance	0.875	0.89	0.862
2 years	OMS less than −2 SD	12 (15.2%)	7 (8.9%)	4 (5.1%)

IUGR: intrauterine growth restriction. EUGR: extrauterine growth restriction.

**Table 4:** Comparison of scalar scores for Inhibition, Memory for names, Memory for designs, and Route finding, and of IQ for RIST, between cases with and without diagnosed clinical neurological disorders.

	Diagnosed Clinical Neurological Disorders (*n* = 19)	Not Diagnosed Clinical Neurological Disorders (*N* = 68)	Est Sig ^#^ (*p*)
RIST (median (IQR))	89 (42)	91 (18)	0.426
Inhibition (median (IQR))	7 (5)	10 (4)	0.002
Memory for names (median (IQR))	9 (7)	10 (4)	0.492
Memory for designs (median (IQR))	10 (3)	10 (4)	0.630
Route finding (median (IQR))	1 (1)	3 (4)	0.010

^#^ Mann–Whitney U-Test.

**Table 5:** Statistically significant differences found in RIST index of different somatometric classification groups.

	Yes	No	Est Sig
IUGR weight Fenton < P10	84 (20)	95 (25)	0.037
IUGR length Fenton < P10	84 (22)	93 (25)	0.027
IUGR length IGW-21 < P10	84 (24)	93 (25)	0.025
IUGR HC Fenton < P10	84 (18)	93 (25)	0.006
IUGR HC IGW-21 < P10	84 (18)	93 (25)	0.047
Static EUGR length Fenton < −2 SD	84 (20)	93 (28)	0.03
Static EUGR length IGW-21 < −2 SD	84 (19)	95 (28)	0.01
Static EUGR HC Fenton < P10	83 (25)	93 (23)	0.018
Static EUGR HC IGW-21 < P10	85 (23)	92 (23)	0.001
Two years HC OMS < −2 SD	70 (28)	92 (21)	0.025

Mann–Whitney U-Test.

## Data Availability

The datasets generated during and/or analysed during the current study are available from the corresponding author on reasonable request.
